# Silver Nano/Microparticles: Modification and Applications

**DOI:** 10.3390/ijms20112609

**Published:** 2019-05-28

**Authors:** Bong-Hyun Jun

**Affiliations:** Department of Bioscience and Biotechnology, Konkuk University, Seoul 143-701, Korea; bjun@konkuk.ac.kr

Nano/micro-size particles are widely applied in various fields. Among the various particles that have been developed, silver particles are among the most important because of their favorable physical, chemical, and biological characteristics [1]. Thus, numerous studies have been conducted to evaluate their properties and utilize them in various applications, such as diagnostics, antibacterial and anticancer therapeutics, and optoelectronics [2,3,4,5,6,7,8]. The properties of silver particles are strongly influenced by their size, morphological shape, and surface characteristics, which can be modified by diverse synthetic methods, reducing agents, and stabilizers [9].

This Special Issue provides a range of original contributions detailing the synthesis, modification, properties, and applications of silver materials, particularly in nanomedicine. Nine outstanding papers describing examples of the most recent advances in silver nano/microparticles are included.

Lee et al. comprehensively described the synthesis of silver nanoparticles by various physio-chemical and biological methods and elucidate their unique properties which are useful for applications such as for developing antimicrobial agents, biomedical device coatings, drug delivery carriers, imaging probes, and diagnostic and optoelectronic platforms [10]. The underlying intricate molecular mechanisms behind the plasmonic properties of silver nanoparticles on their structures, potential cytotoxicity, and optoelectronic properties were also discussed.

Several innovative silver-based nanomaterials have been introduced in bio-applications. Kang et al. reported a functionalized β-cyclodextrin (β-CD)-immobilized silver structure as a drug carrier [11]. Synthesized β-CD derivatives, which have beneficial characteristics for drug delivery including hydrophobic interior surfaces, were immobilized on the surface of silver-embedded silica nanoparticle to load doxorubicin (DOX). DOX release and its effects on cancer cell viability were studied. Liu et al. reported polydopamine (PDA)-assisted silver nanoparticle self-assembly on a sericin (SS)/agar film with potential wound dressing applications [12]. They prepared an SS/agar composite film, and then coated PDA on the surface of the film to prepare an antibacterial silver nanoparticle-PDA-SS/agar film, which exhibited excellent and long-lasting antibacterial effects. Radtke et al. studied silver ion release processes and the mechanical properties of surface-modified titanium alloy implants [13]. Dispersed silver nanoparticles on the surface of titanium alloy (Ti_6_Al_4_V) and titanium alloy modified with a titania nanotube layer (Ti6Al4V/TNT) as substrates were prepared using a novel precursor with the formula [Ag_5_(O_2_CC_2_F_5_)_5_(H_2_O)_3_] and may be suitable for constructing implants with long-term antimicrobial activity. The properties of silver nanoparticles have been widely studied, including by surface-enhanced Raman scattering (SERS). Pham et al. reported the control of the silver coating on Raman label-incorporated gold nanoparticles assembled as silica nanoparticles for developing a strong and reliable SERS probe for bio-applications [14]. A SERS-active core Raman labeling compound shell material based on Au–Ag nanoparticles and assembled on silica nanoparticles can be used to solve signal reproducibility issues in SERS.

Humans and the environment are becoming increasingly exposed to silver nanoparticles, raising concerns about their safety. Liao et al. focused on the bactericidal and cytotoxic properties of silver nanoparticles [15]. Silver nanoparticles have been reported to be toxic to several human cell lines. In their paper, the state-of-the-art of applications in antimicrobial textile fabrics, food packaging films, and wound dressings of silver nanoparticles in addition to the bactericidal activity and cytotoxic effect in mammalian cells are presented. Fehaid et al. conducted an in-depth study of the toxicity of the size-dependent effect of silver nanoparticles [16]. Since tumor necrosis factor α (TNFα) is a major cytokine that is highly expressed in many diseased conditions, the size-dependent effect of silver nanoparticles on the TNFα-induced DNA damage response was studied. Yan et al. focused on the impacts of silver nanoparticles on plants [17]. They summarized the uptake, translocation, and accumulation of silver nanoparticles in plants and described the phytotoxicity of silver nanoparticles towards plants at the morphological, physiological, cellular, and molecular levels. The current understanding of the phytotoxicity mechanisms of silver nanoparticles were also discussed.

Silver particles can also be used as ink. Mo et al. summarized silver nanoparticle-based ink with moderate sintering in flexible and printed electronics [18]. They developed methods and mechanisms for preparing silver nanoparticle-based inks that are highly conductive under moderate sintering conditions and applied the ink to a transparent conductive film, thin film transistor, biosensor, radio frequency identification antenna, and stretchable electronics. The authors summarized their perspectives on flexible and printed electronics.

Silver nano/microparticles are emerging for use in next-generation applications in numerous fields including nanomedicine. The potential benefits of using silver as a prominent nanomaterial in the biomedical and industrial sectors have been widely acknowledged. This Special Issue highlights outstanding advances in the development of silver nano/microparticles as well as their modification and applications.
